# Understanding barriers to and strategies for medication adherence in COPD: a qualitative study

**DOI:** 10.1186/s12890-022-01892-5

**Published:** 2022-03-19

**Authors:** Jacqueline O’Toole, Meera Krishnan, Kristin Riekert, Michelle N. Eakin

**Affiliations:** Division of Pulmonary and Critical Care, Johns Hopkins University School of Medicine, Baltimore, USA

**Keywords:** Patient experience, Barriers to care, Access to care, Adherence, Routine

## Abstract

**Background:**

Medication adherence in chronic obstructive pulmonary disease (COPD) is low, though not enough is known about the factors that affect adherence in COPD. This study uses qualitative methods to understand the patient perspective on facilitators and barriers to medication adherence in COPD as well as patient-reported strategies for self-management of disease.

**Methods:**

Semi-structured interviews were conducted with 30 individuals (n = 30). Transcripts were analyzed using iterative qualitative coding by 2 independent coders, and codes were categorized using thematic analysis.

**Results:**

Challenges with adherence reported were gaps in understanding, forgetfulness of the patient, physician availability, cost navigation, and overcoming substance use. Most commonly, the financial burden of COPD medications caused patients to source other countries to obtain medications, rely on sample medications collected during doctors’ visits, and to alter medication dosage and frequency to extend the length of a prescription. Tools and resources reported by patients to support self-management of COPD included pharmacist assistance, physician office information, and community resources. Individuals further reported that the use of logs or diaries to track medication usage, visual or temporal cues to take medications, and support from family members were helpful in promoting adherence to their COPD medication regimen.

**Conclusions:**

Medication adherence in individuals with COPD is affected by challenges with self-management of disease and financial burden of medications. However, patients reported multiple tools and resources to support adherence. Physician recognition of these factors impacting self-management, as well as awareness of strategies to promote adherence and manage disease, may improve patient outcomes.

**Supplementary Information:**

The online version contains supplementary material available at 10.1186/s12890-022-01892-5.

## Introduction

Chronic obstructive pulmonary disease (COPD) is a highly prevalent disease worldwide and contributes to high mortality and cost within the medical system [1–3]. Adherence to medication regimens in COPD has been notoriously low, with reports of 15–30% adherence [4–7]. Low adherence in COPD is associated with higher healthcare costs, increased hospitalizations, and worse disease control [8]. In recent years, the European Union has made adherence practices a priority in health care, particularly in reference to COPD [3]. Low adherence stems from many factors, including suboptimal communication, financial burdens, time intensive regimens, comorbid diseases, and personal factors that affect the ability to self-manage disease [9–11].

Achieving good adherence involves a process including the initiation, implementation, and persistence of a treatment plan [12]. In COPD, this means obtaining and taking the first inhaled dose followed by obtaining and taking refills of the medication over time. To accomplish this, individuals must have the ability to understand, obtain and correctly administer medications on an appropriate schedule, which presents many challenges. One such challenge is that individuals with COPD often have comorbid diseases, resulting in a larger burden of care than managing an isolated disease [10, 13]. In addition, inhaled medications tend to be expensive and with varying insurance coverage making access to maintenance of therapy challenging [10, 13]. Patients taking medication for COPD are often forced to choose where to allocate financial resources each month among other medical and daily living needs [10].

The quantity and variety of different inhaler delivery devices for COPD pose unique challenges for self-management, particularly in an aging population with dexterity and visual limitations. There are at least seven inhaler delivery devices available, meaning individuals with more than one prescribed inhaler may have more than one device to learn [14]. Despite guidelines recommending inhaler teaching, the process of prescribing inhaled therapies and ensuring proper usage among affected patients is often inconsistent and unclear [15, 16]. This raises a technical challenge for patients and highlights the importance of good communication surrounding medication plans specifically with one’s care team. The strength of patient-doctor interactions is pivotal for supporting adherence to inhalers [17].

Though it is well-established that adherence in COPD is low, little is known regarding individual perspectives on these challenges and what resources and tools people with COPD view as most important to assist in adherence to COPD medication plans. Through the National Health Service in Great Britain, informative data at a population level documenting the presence of general patient experiences with COPD are available. However, exploration of such experiences and how they pertain to self-management of disease are lacking [18]. With so many factors contributing to adherence, it is important to understand what people with COPD value and experience. The goal of this study is to explore the patient perspective regarding medication adherence in COPD and gain insight to facilitators and barriers to medication adherence as well as patient-reported strategies for maintaining adherence. The patient perspective regarding medication adherence in COPD management will be informative to future efforts to target initiatives to improve adherence, a necessary step in improving COPD outcomes.

## Methods

### Participants

A convenience sample of individuals enrolled and followed in the ongoing Medication Adherence Research in COPD (MARC) Study was contacted to participate in semi-structured telephone interviews. The MARC study is a longitudinal observational study investigating adherence behaviors in individuals with COPD and COPD outcomes. All study procedures, including the need for verbal informed consent and the script for consent, were approved by the Johns Hopkins Institutional Review Board (IRB).

Participants met the same inclusion criteria as the MARC study (≥ 40 years old, physician diagnosis of COPD, GOLD stage II-IV disease, and prescribed a long-term controller medication). Individuals also could not have a patient-provider relationship with any members of the research team. MARC participants were invited to be contacted to participate during study follow up phone calls or visits. Interested individuals were scheduled for an interview in the order in which they agreed to learn about this sub-study and oral consent was obtained prior to initiation of audio recording and other study procedures in accordance with IRB. Participants were reimbursed for their time.

Demographic information including educational history and cognitive impairment assessment data was collected as part of the MARC study. Cognitive impairment was assessed using the Montreal Cognitive Assessment (MoCA), a validated screening test for cognitive impairment with scores < 26 indicating mild cognitive impairment [19]. Additional items including working and independent living information were obtained prior to interview.

### Interviews

Interviews were conducted using a semi-structured interview guide (Additional file 1). Following a literature review, the guide was discussed and developed with the help of experts in behavioral therapy and qualitative research (MNE, KR). Interviews were designed to explore strategies, barriers and processes related to self-management of COPD and communication during a doctor’s office visit. The semi-structured interview guide allowed for inclusion of follow-up questions based on individual interview responses. Ongoing review of interview transcripts allowed adaptation of subsequent interviews to explore themes as they emerged. The interview guide was updated by JO and MNE to include prompts for facilitation of more in-depth discussion of emerging themes. Questions were modified if necessary for clarity.

Individuals completed a semi-structured interview that was audio-recorded. All interviews were conducted by a female research pulmonary fellow (JO). She was identified to participants by first name as a research fellow with little emphasis or reference to her clinical practice, to avoid potential bias of interviewees’ responses based on their own experiences with physicians.

Audio recordings of interviews were transcribed by a confidential transcription company specializing in research transcripts and returned for review. Transcriptions were completed in batches of 4–6 to allow for review of interview content to ensure themes were being explored.

### Analysis

Content analysis of interview responses were conducted using an inductive approach [20]. Following the completion of 10 interviews, MNE and JO independently reviewed transcripts for identification of themes and these were used to develop a code book. An additional five interviews were reviewed for further emerging themes to add to code book. Once no new themes emerged on three consecutive interviews, concept saturation was felt to be met [21]. Transcripts were subsequently coded with NVivo 12 software using the developed code book. Interviews were coded by two independent coders (JO and MK) with any major discrepancies adjudicated by a third party (MNE). An overall kappa was calculated to be 0.75 signifying good agreement [22]. Codes were then categorized into larger themes using thematic analysis.

## Results

**Fig. 1:**
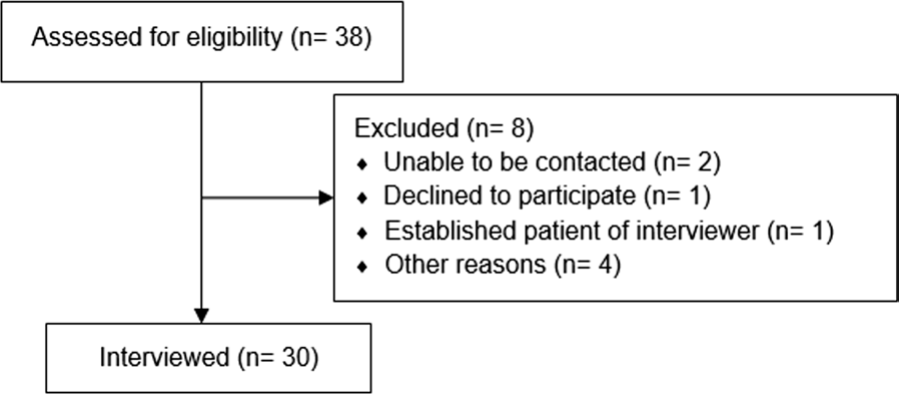
CONSORT diagram for interview participants

Of the 55 individuals enrolled in the MARC study between April 2017 and April 2019, 38 individuals were sequentially approached to participate in this sub-study with 37 of those agreeing to be contacted at the time the goal of 30 interviews was reached and recruitment stopped. Of the 37, two individuals were unable to be contacted after multiple attempts, 1 individual was excluded because of prior patient-provider relationship with the interviewer and 30 individuals completed interviews (Fig. 1). Once thirty interviews were completed, individuals were no longer approached to participate. Thirty individuals with COPD (mean age 70 ± 8.3 years, 43% men, 73% white, 93% high school degree or higher education) were interviewed by telephone. There were 11 (37%) living alone, 17 (57%) retired, 7 (23%) on disability, and 8 (27%) had been hospitalized within the 12 months prior to interview. At least mild cognitive impairment (< 26 MoCA) was seen in 53% of interviewees (Table 1). Interviews lasted a mean (SD) of 26.9 (7.6) minutes.

**Table 1:** Baseline characteristics for interview participants

	N = 30
Age (mean ± SD)	70 ± 8.3
Female	17 (57%)
Race	
White	22 (73%)
Black or African American	7 (23%)
Multiracial	1 (3%)
Mild cognitive impairment or worse n (%)	16 (53%)
High school degree or higher education	28 (93%)
Lives alone	11 (37%)
Lives with a roommate or spouse	13 (43%)
Retired	17 (57%)
Disabled	7 (23%)
Hospitalized in the prior 12 months	8 (27%)

Three major concepts regarding self-management emerged from interviews: (1) Challenges with self-management of disease, (2) Financial burden of medications, and (3) Tools and resources utilized to support adherence.

### Challenges with self-management of disease

Challenges reported with self-management included gaps in understanding, forgetfulness of the patient, physician availability, cost navigation, and overcoming substance use (Table 2). Many individuals reported forgetfulness as a barrier to medication administration, particularly medications due more than once a day. One individual reported he “may forget to take the evening medicines because I fell asleep.” (66M) Another referred to difficulty with dosing frequency, “for a while I was taking medications twice a day. And that did get a little confusing, especially when I started to feel better, and I would sometimes forget about taking my afternoon medication.” (67M).

**Table 2:** Challenges with self-management of disease

Themes	Illustrative quotes
Gaps in understanding	I didn't know that not taking the medicine made it worst. 59F
	Sometimes I'll forget the evening one. Not that I forget. I just don't feel like getting up to get it or don't feel like I necessarily need it. 68F
Navigating costs	I just changed to a new plan so I wouldn't be in a donut hole next year. But when you change plans, sometimes it's two or three months before you can get—God forbid. 59F
	The insurance company I was using no longer would cover it. So I had to switch from the [XXX] to something. So I've been through this a couple of times. 56F
	I have [XXX insurance], and they won't let me get a lot of stuff. We first tried with the patches [for tobacco cessation], and it didn't seem to work for me. Then he prescribed me gum and [XXX insurance] wouldn't pay for it, so I couldn't get that. 61M
	There's been a couple of times I've asked about medicines, and I've been told that the VA doesn't support it, so I can't get it. 70M
Barriers to access- physician availability	I avoid going to the doctors because I don't think they're paying real attention, and they're on a timeframe. 69F
	I dropped Dr. [XXX] because whenever I have any questions or anything, I call, I couldn't get her. 84F
Forgetfulness	I have to try to remember to take my medicines sometime in the daytime because some of them is like, "Eat with food. Eat after food. Eat before meals," and I have to realize that so that I don't forget because it's like nine pills. 69F
	For some reason, I don't always remember the evening one only because I just don't remember. I'm just not used to taking meds that late at night and half the time, it's after I get in bed and I think, "Oh, I forgot to take it," but I try. 80F
Barriers to adherence: substance use	
	I hate to say it but because I receive Suboxone, I think I'm just treated like a druggie. Hey, she comes once a month. …I feel like I'm one of the druggies. Or the other patients who are seen on days other than Thursdays get the better attention because they've earned it and they're not full of drug addicts. 58F
	For instance, there's one medicine I have to take in the afternoon …but I do tend to start drinking right after lunch…if I'm too inebriated or too influenced, I won't take that pill. And I'll just wait until I'm more sober. 67M

Frustration with inhalers and lack of guidance contributed to challenges at home. This was exemplified by one individual expressing that his provider “didn’t give me any instructions how to use it [inhaler]” (65M). Other individuals expressed lack of physician follow-up on inhaler technique despite experiencing difficulties learning or remembering how to properly administer their inhaled medications. One individual reported she does not use a spacer when in public because “I find it very embarrassing… but I just won't take that chamber out of my purse and use it.” (62F) Drug use was also reported as a barrier to self-management of disease both in regards to associated stigma when seeking care and in regards to taking medication appropriately, as substance use caused some to report they “didn't care whether I was doing the right thing or not with my medications.” (56F) (Table 2).

### Managing the financial burden of medications

Navigating costs of medication and insurance rules as a challenge was reported, in particular starting a medication because of a coupon or free sample only later to find out “my insurance company wouldn’t pay for that.” (70M) Individuals also reported different techniques to deal with the burden of cost of inhaled therapies (Table 3). Some individuals reported they circumvented costs by going “over the border into Canada” (60F) to obtain affordable medications. Others had success with self-advocacy and writing to pharmaceutical companies to obtain waivers of cost for medications. Still, some reported adjusting medication dosing and frequency to extend the length of a prescription in an attempt to save costs, resulting in medication underuse. While others reported overuse of medications in an attempt to recoup costs already spent, reporting “He had told me that I can stop taking the XXX, but actually, I still take the XXX because I got three canisters of it, and I paid my money for it, so I just take both of them… it's so expensive. I don't want to just throw that away.” (70M) when a new inhaler was prescribed soon after he had refilled his prior inhaler. Several individuals reported the use of sample medications from doctor appointments in order to avoid buying prescriptions at the pharmacy. As one patient describes, “Every time I go, I ask for samples because they're so expensive, even with my co-pay.” (60F) An alternative strategy for addressing this issue included making it a point to discuss cost with doctors to request medicine changes “because it’s cheaper” (72F) for certain inhalers as compared others.

**Table 3:** Managing the financial burden of medications

Themes	Illustrative quotes
Medication misuse	I was taking one puff maybe in the morning and maybe a couple days later I would take another puff … And the reason I was doing that was because—like a whole lot of other people who don't take their medicine right—the cost of the medication. 84F
Outsourcing	I needed these medicines. And I couldn't afford them, so I ordered them from Canada…I understood there was a chance that there still could be something foul in them, but I took the chance. I couldn't afford them otherwise. 70M
Collaboration with care providers to circumvent cost challenges	I found out that instead of costing me 47 a month for the rest of the year, it's going to cost me about 150 because I'm in [retirement?] home. In fact, I was calling the doctor today to have her to put me back on [XX] because it's cheaper. 72F
	I discuss it with my doctors. And sometimes they say, "Well, unfortunately, things are what they are and we really can't change them…" But they, I mean, they're sympathetic, but. And sometimes I'll ask, "Is there one I can get that isn't as expensive?" 80F
	My doctor gives me an awful lot of samples of this, and that helps me a great deal. 79F
Cost savings strategies	I wrote to [inhaler company] and I contacted them because I was in a donut hole. And they gave me a year free. 72F
	I paid dearly for my medicine so I didn't want to just stop … He changed my medicine next week, and I just got 30 days supply. I would finish my 30 days supply because otherwise I'm throwing my money out the window. 59F
	At a certain age, I realized that I needed a secondary health plan…to go along with my Medicare and my Medicaid, I needed a helper. 69F

### Tools and resources to support adherence

Pharmacist assistance, physician office information, and community resources were among the tools and resources reported to aid in adherence to a patient’s COPD medication regimen (Table 4). One individual reported that her “pharmacy helps me a lot with my medicine.” (54F) Another said that summaries from her physician’s office are something she uses for reference of her disease control and she “keep[s] a copy of my results from year to year” marking stability of disease. (71F) Community resources included support groups through the American Lung Association and local health fairs.

**Table 4:** Tools and resources to support adherence

Themes	Illustrative quotes
*Strategies*	
Logs of medications	I started a little diary program and documented when I took what. Now, I pretty much have it down to a science. 60F
	I couldn't remember everything. So my boss, my wife told me to make a list, check it twice, that kind of thing. 69M
Consistent location for medications to be kept	Keeping them in groups, definitely it does [help]. If you have one medication in one place and one medication in another room, that wouldn't work for me. 70F
	I have everything in my one nightstand, the bottom drawer. All my medications. 56F
Consistent routine	I have an alarm set on my phone for 8:30 every morning. When I turn that off, I get up and I go to the meds immediately. It takes minute and a half, two minutes, and it's over for the day. 72F
	My Spiriva sits right near my coffeemaker, okay? So as I'm making the coffee, I'll take two hits off of Spiriva. Then I get my coffee. 62M
Family and support network	I am not allowed to go alone. My wife will not let me go to a doctor's visit by myself. It's helpful from the standpoint that if I miss something—and as the older I get, I usually do—or I don't understand something, or I forget to ask a question, my wife does it for me. 69M
	I have four children that are very good to me and if I run short, if I said, "Listen, I can't afford my meds," they'd give me a check for 50 bucks right away, and, "Go get it, Mom." 80F
Community resources	I started going to a support group. And then I started pushing for one in Frederick, and then they started one that's at Frederick hospital, a Better Breathers Club. 70M
	I did go to a LUNG FORCE expo or something. American Lung Association did a seminar in Baltimore last year, I went to that, which was somewhat helpful. 60F
	The insurance company, they are really totally professional… And they're very polite, very professional, very helpful. 58F
Physician Office resources	I asked for a copy of everything that he did that day because I'm 60 and I don't remember medical terms. 59F
	All the medicines I get from the VA come with a very comprehensive fact sheet on them…Tells you how to take them, what they do, what the side effects are. 66M
Computerized resources	MyChart's good for [follow-up]. I followed up with my doctors with a couple things on MyChart. It might take a day or two to get back, but they usually do. 65M
	I can go onto MyChart and change and add prescriptions that I'm taking. And when he prescribes something, it goes in there. 56F
	I get all the details, I go onto the Internet and try to pull up the information as well. 55M
Pharmacy resources	If I really, really wanted the answers, which I—that's how I found out I was using the inhaler too much was I disregarded their [doctors'] sheets and went to these sheets from [Pharmacy]… It's kind of like [Pharmacy] would give you a book, and the doctor would give you a short story. 55M
	I've learned more when I went down to the pharmacy and got it. They give you that little paper along with it, and I sat there and read everything about it. 61M

Tools for ensuring medications were taken included the use of logs or diaries to track when medications were administered. Incorporating medication into the morning routine helped some, one individual saying “checking your email and taking your meds, that’s what I do [each morning].” (60F) Others rely on visual cues, choosing to store inhalers in specific locations as they are used during the day. As one patient expressed, “if it’s at 10 o’clock in the morning, if it’s still on the dining room table, I know I haven’t taken it.” (74M).

Additionally, many patients cited social support as a facilitator to adherence to their prescribed medication regimen. When speaking about the importance of having support during an office visit, one individual remarked “if someone were to go with me, that person could take notes…Because sometimes what we're hearing and how we interpret it are two different things.” (62F).

## Discussion

This study highlights the perspectives and experiences from people with COPD as it relates to their disease management. Individuals identified facing many barriers to medication adherence, including inhaler visibility, disease stigma, medication costs, and challenges with patient-provider communication. Participants also provided a number of strategies they used to help manage medications including maintaining a daily routine, engaging in patient empowerment by providers and pharmacists, and family accompaniment to doctor visits.

One of the primary strategies for medication adherence was the maintenance of a daily routine with an established time and location for taking medications. As such, disruption of this daily routine contributed to failure to take medications as prescribed. Individuals also reported the second dose of medication each day was challenging to successfully take. These observations suggest that people with COPD who are unable to establish a regular daily routine may struggle to take their medications on time until these external factors have stabilized. Incorporating medication schedules into a daily routine with anticipatory guidance for how to handle late doses and doses due when away from the home is an area for intervention.

Outside of the home, participants reported that the visibility of inhalers and inability to hide their taking of medication affected their willingness to take medication in public. Stigma associated with disease has been reported previously to impact quality of life and medication adherence in COPD [23]. Prior data on people with COPD perspectives and oxygen utilization also demonstrated hesitancy to use a treatment modality so visible to others [9]. Providers have an opportunity to discuss openly these concerns and work with each patient, potentially identifying support to improve upon stigma perceived by patients with COPD.

Addressing challenges with cost of medication was noted by the participants as one of the most salient themes. Most commonly, discussion of cost directly related to underuse of medication. However, on more than one occasion, cost was a causative factor in promoting overuse of medication or use of duplicate medications. Personal strategies to address the high cost of medications, including using sample medications from doctors’ visits and traveling to other countries to obtain medications, were commonly reported by people with COPD. Given the number of inhalers in each drug class available, providers may be able to address this challenge by working with patients and pharmacies to prescribe the most financially accessible medication with appropriate efficacy for an individual. Working with people with COPD to find inhalers that are not cost-prohibitive can help improve the likelihood a person is able to obtain the medication [10]. Challenges with medication adherence due to cost can further be addressed by utilization of combination inhalers where appropriate and streamlined dosages to once daily. Although individuals with COPD who live outside of the US may not have as many challenges related to cost of medications, the EU congress report still reported that medications can be switched to appease insurance requirements which can be very burdensome for patients [3].

Furthermore, challenges with patient-provider communication were reported frequently as barriers to medication adherence. Such challenges included confusion regarding what the provider is asking of the patient, insufficient or confusing instructions regarding inhaler use and techniques, and an inability to communicate with providers outside of scheduled appointments. Lack of clear and consistent patient-provider communication may also pose a barrier to discussion of cost concerns. These challenges contributed to gaps in understanding of the disease process and indications for therapies, which in turn may have affected adherence, according to some individual’s reports.

A common sentiment expressed relating to personal motivation for COPD management was the importance of feeling heard during doctor visits, which contributed to confidence in providers and prescribed care regimen. Communication with community pharmacists has previously been noted to improve self-confidence in use and may increase adherence [24], as does physician follow-up regarding technique and usage of inhaled devices [25, 26]. Pharmacy involvement is particularly important for inhaler usage given previous data showing physicians lack nuanced understanding themselves of appropriate use of all inhalers [27, 28].

Accompaniment by family at physician visits was cited by participants to have a positive impact on information retention by patients which in turn may improve adherence to prescribed medications. Specifically, patients felt that the presence of a family member at visits who could make comments, ask questions, or take notes enhanced their ability to communicate with their provider during the visit and allowed for more detailed recall of provider instructions after the visit when taking their medications. This is particularly notable in the context of the burden of cognitive impairment in individuals with COPD, both previously demonstrated and demonstrated in this cohort of people with COPD with 53% having at least mild cognitive impairment [7, 29]. Interestingly, similarities to previously documented factors affecting asthma management were observed, including that individuals valued clear communication with physicians as facilitators to disease control and behaviors were influenced by the cost of inhalers [30]. This work will be informative for the development of interventions aimed at improving self-management in COPD.

A strength of the semi-structured interview format used for data collection in this study is the ability to capture experiences and priorities that multiple choice or quantitative measures cannot access or may not properly represent. However, one limitation to phone interviews is the inability of the interviewer to observe non-verbal cues, which may contribute to understanding of interview responses.

Finally, when interviewing and subsequently coding qualitative data, there is an inherent bias of the interviewer that requires framing and acknowledgement. The practice of utilizing memos and reflection during the coding process served to mitigate some of these biases [31]. Although efforts were made to include a diverse sample (e.g., approximately 25% non-white), our results may not generalize to all individuals with COPD given that we were restricted to one geographic location. Qualitative analysis itself is not generalizable as it represents specific experiences by individuals, though by interviewing to the point saturation of themes is reached one can make inferences into thematic trends with confidence [31, 32].

## Conclusion

Many factors impact medication adherence in people with COPD. The significance of such factors from a patient perspective is imperative to understand when working to improve adherence. The results of this study shed light on challenges commonly faced in medication adherence, strategies to reconcile with the financial implications of COPD medication regimens, and tools to assist with medication regimens, as reported by people with COPD. The recognition of such factors effecting adherence is prerequisite to the delivery of collaborative care and improvement of self-management of disease in COPD. By acknowledging and discussing tools commonly used by people with COPD for disease self-management and promoting open communication between patients and their care team, a patient-centered approach for adherence to a COPD medication plan can be enacted.

## Supplementary Information

**Additional file 1.** Patient participant semi-structured phone interview guide.

## Data Availability

The datasets generated during and analyzed during the current study are not publicly available due to the possibility that containing that could compromise the privacy of research participants as well as occasions of institutional and pharmacologic references within interview transcripts. Data are available from the corresponding author on reasonable request.

## Abbreviations

COPD: Chronic Obstructive Pulmonary Disease
MARC: Medication Adherence Research in COPD
IRB: Institutional Review Board
GOLD: Global Initiative for Chronic Obstructive Lung Disease
MoCA: Montreal Cognitive Assessment
