# Supplementary material for: Understanding barriers to and strategies for medication adherence in COPD: a qualitative study
Source: BMC Pulm Med. 2022 Mar 19;22:98. doi: 10.1186/s12890-022-01892-5 (PMC8934480; doi:10.1186/s12890-022-01892-5)
Supplement: Supplementary file 1 — Additional file 1. Patient participant semi-structured phone interview guide. [file 12890_2022_1892_MOESM1_ESM.docx]

Patient Participant Semi-Structured Phone Interview Guide

I am now pressing play on the recorder and I will be recording the rest of our discussion. Please stop me if you have questions or if you wish to stop recording for any reason. As we discussed during the informed consent, the goal of this study is to talk to people with COPD and learn more about how you keep track of medicines and how the time you spend at a doctor’s visit helps or does not help this.

I want to learn about your experiences to help improve communication about medicines. These questions will help us understand this better and hopefully help make these things easier in the future, there are no right or wrong answers.

**Icebreaker:**

Please tell me what medicines you take for COPD.

**Theme: Office communication around medication initiation**

1. To start, when I ask about your doctor, I mean the doctor who takes care of your COPD Please tell me about a time your doctor gave you a prescription for a new medicine for your COPD.
   1. Probe if not discussed: What information did your doctor give you?
   2. What did he/she say about what the medicine was for? What can you remember?
   3. Probe if not discussed: What, if any, directions did he/she give you?
   4. Probe if not discussed: What, if any, questions did you ask?
   5. Wat can you remember?534-35 give you? for COPD
      rs.l livingindependently prior to critical illness. or quantitative measures, 4hat has been helpful for you to hear when getting new medicines?
   6. If a friend of yours was getting a new medicine for COPD, what information do you think their doctor should tell them?
2. Now, thinking back to when you got an inhaler for the first time, how were you told or shown how to use the inhaler, if you were?
   1. How does the doctor or office check on your inhaler technique over time?
   2. What, if anything, does the doctor or office staff do to check if you are having any problems with your inhaler? (For example: What if the inhaler seems broken? Misfires?)
3. Has your medicine ever been changed because of insurance? If so, can you please tellme more about that?
   1. How were you told or shown to use the new medicine?
   2. What questions did you have?
   3. What did your doctor say about the change?
4. Please tell me about a time when the cost of a medicine has played a role in your ability to get the medicine?
   1. Have you ever missed medicine doses because you could not afford to get the medicine?
   2. What have you and your doctor talked about regarding the cost of medicines?
      1. How does the cost of medicines impact you?

**Theme: Patient-provider medication reconciliation**

1. When you are in the doctor’s office, how are your medicines reviewed?
   1. Probe if not discussed: Who is involved with this? If it’s more than one person, let me know all of them.
   2. What if you cannot remember the medicine’s name?
   3. If you stopped or increased usage of a medicine how would your doctor find this out?
2. Ask if not already mentioned or discussed: Can you tell me about a time when it was confusing to know what medicine to take?
   1. Probe if not discussed: How did you work through this confusion?
   2. What helped you so you could avoid this from happening again?

**Theme: Education and resources utilized in office visits**

1. What, if anything, do you get from your doctor’s office to help you know how to take your medicines?
   1. What type of education do you get from the doctor’s office on the medicines? Or,what does the doctor or office do to let you know how to best use or take your medicine?
   2. Probe if not discussed: Do you get any paper or summary of your visit when you leave? If yes, what, if anything, do you find helpful about the summary?
   3. Probe if not discussed: Do you bring someone with you to office visits? What if anything is helpful about that?
      1. What caused you to start doing that?
   4. Would anything make remembering what the doctor goes over easier?

**Theme: Patient process of adherence**

1. Now I would like to talk about what happens after you leave the doctor’s office. Changes in work schedule, travel, and time of year can all affect your daily routine and make it hard to take medicines. Some patients say they have trouble remembering to take their medicines (pills and inhalers). How often do you have trouble remembering to take your medicines?
   1. In a normal week how do you keep track of your medicines for COPD?
   2. How do you handle it when schedules change?
2. I know it is hard to take medicines exactly as prescribed, when you take medicines different than prescribed, how does the doctor know how often you take your medicines?
   1. Follow up on the “only if I tell her” with “are you likely to tell her?”

**Theme: Identifications of barriers to adherence**

1. Have you ever had difficulty remembering or keeping track of medicines that you are on? If yes, can you tell me about that?
2. What systems do you have to help you remember?
   1. Probe: if you use a pill sorter, how do you adjust for the inhalers that don’t fit into that?
   2. Probe: how did you decide to use X? (if mention a memory aid such as writing down/keeping computer log, etc). Where did you get that idea?
   3. Probe: If you do have a strategy, how do you update your list/pill sorters when medicine lists change?

This is the end of our interview. Thank you for talking to me. If you would like to continue talking to someone about your COPD you can contact the Better Breathers club or your doctor’s office for information on other COPD groups near you.
